# Analysis of DNA Methylation Patterns Associated with In Vitro Propagated Globe Artichoke Plants Using an EpiRADseq-Based Approach

**DOI:** 10.3390/genes10040263

**Published:** 2019-04-01

**Authors:** Elisa Cerruti, Cinzia Comino, Alberto Acquadro, Gianpiero Marconi, Anna Maria Repetto, Anna Barbara Pisanu, Roberto Pilia, Emidio Albertini, Ezio Portis

**Affiliations:** 1 Department of Agricultural, Forest and Food Sciences, Plant Genetics and Breeding, University of Torino, 10095 Grugliasco, Italy; elisa.cerruti@edu.unito.it (E.C.); alberto.acquadro@unito.it (A.A.); ezio.portis@unito.it (E.P.); 2 Department of Agricultural, Food, and Environmental Sciences, University of Perugia, 06121 Perugia, Italy; gianpiero.marconi@unipg.it (G.M.); emidio.albertini@unipg.it (E.A.); 3 Agris Sardegna—Agenzia Regionale per la Ricerca in Agricoltura—Servizio Ricerca sui Sistemi Colturali Erbacei, 09123 Cagliari, Italy; amrepetto@agrisricerca.it (A.M.R.); abpisanu@agrisricerca.it (A.B.P.); rpilia@agrisricerca.it (R.P.)

**Keywords:** globe artichoke, in vitro culture, DNA methylation, EpiRADseq, epigenetics, somaclonal variation

## Abstract

Globe artichoke represents one of the main horticultural species of the Mediterranean basin, and ‘Spinoso sardo’ is the most widespread and economically relevant varietal type in Sardinia, Italy. In the last decades, *in vitro* culture of meristematic apices has increased the frequency of aberrant plants in open-field production. These off-type phenotypes showed highly pinnate-parted leaves and late inflorescence budding, and emerged from some branches of the true-to-type ‘Spinoso sardo’ plants. This phenomenon cannot be foreseen and is reversible through generations, suggesting the occurrence of epigenetic alterations. Here, we report an exploratory study on DNA methylation patterns in off-type/true-to-type globe artichoke plants, using a modified EpiRADseq technology, which allowed the identification of 2897 differentially methylated loci (DML): 1998 in CG, 458 in CHH, and 441 in CHG methylation contexts of which 720, 88, and 152, respectively, were in coding regions. Most of them appeared involved in primary metabolic processes, mostly linked to photosynthesis, regulation of flower development, and regulation of reproductive processes, coherently with the observed phenotype. Differences in the methylation status of some candidate genes were integrated with transcriptional analysis to test whether these two regulation levels might interplay in the emergence and spread of the ‘Spinoso sardo’ non-conventional phenotype.

## 1. Introduction

Globe artichoke (*Cynara cardunculus* L. var. *scolymus*) is a diploid (2n = 2× = 34), mostly cross-pollinated species native of the Mediterranean basin, with a genome size of ~1.07 Gbp [1]. It is a perennial crop belonging to the Asteraceae family, mostly cultivated for its edible immature inflorescence (*capitulum*), but also exploited as a source for the production of nutraceutically and pharmaceutically active compounds [2,3,4,5,6] as well as of biofuels and oil crop [7,8,9,10]. The ‘Spinoso sardo’ is the most widespread and economically relevant varietal type in Sardinia (Italy).

**Figure 1:**
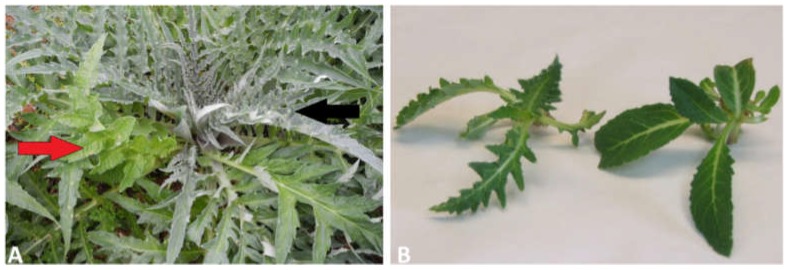
‘Spinoso sardo’ plants showing aberrant phenotype. (**A**) Example of an aberrant ‘Spinoso sardo’ plant in field, showing branches with both standard true-to-type (red arrow) and off-type (black arrow) leaves. (**B**) In vitro plantlets showing off-type (on the left) and true-to-type (on the right) leaves.

In globe artichoke, the traditional propagation method, based on the use of underground dormant buds (‘*ovoli’*) and vegetative offshoots (‘*carducci’*), gives rise to some agricultural issues such as plant heterogeneity, multiplication low rate, and disease transmission [11,12]. Globe artichoke virus-free propagation via in vitro culture of meristematic apices [13,14,15,16] has been well-established in order to preserve the quality of these élite plants. However, in recent years, breeders observed a dramatic increase in the appearance of an aberrant phenotype after open field transplantation of in vitro germinated ‘Spinoso sardo’ plantlets (Figure 1). In field, different branches of the same plant manifest two highly distinguishable phenotypes: the standard early flowering true-to-type, showing large and open leaves, and the late flowering off-type characterized by narrow, darker, and septate leaves.

The phenotypic variability observed among regenerated plants, commonly known collectively as ‘somaclonal variation’, has a negative agronomical impact as it severely reduces the genetic uniformity required for the maintenance of ‘Spinoso sardo’ genotype. In fact, neither ‘Spinoso sardo’ plants carrying those alterations nor heads produced by these branches can be commercialized as a protected designation of origin (PDO) product ‘Carciofo Spinoso di Sardegna’, lacking the desirable élite traits. As these events severely affect the Sardinian agricultural economy, preliminary studies were conducted in order to verify if some biological factors (e.g., subculture number, explant size, and age) might play a role in triggering such a phenotypical aberration. However, the molecular determinants of off-type phenotypes remained far from being elucidated [17].

This phenomenon might be associated to both gene mutations and changes in epigenetic marks [18]. The off-type appearance is unpredictable and reversible through globe artichoke progenies, suggesting that epigenetic mechanisms, rather than genetic mutations, might be triggered during the observed phenotypical rearrangement. Epigenetics refers to heritable changes in patterns of gene expression that occur without alterations in DNA sequence. Epigenetic mechanisms involve covalent modifications of DNA and histones, which affect transcriptional activity of chromatin [19]. Multiple aspects of plant development, including flowering time, stress response, and morphological changes, are directly or indirectly modulated by epigenetic marks, most of which are related to DNA methylation [20,21,22,23,24]. In plants, DNA methylation occurs in the symmetric contexts CG and CHG (H = C, A, or T), and the asymmetric context CHH [19]. CG methylation is maintained through hemi-methylated CG sites, which are complementarily added to the un-methylated newly synthesized DNA strand during DNA replication, whereas CHG and CHH methylation are established and/or maintained by self-reinforcing loops [25].

Although changes in DNA methylation may spontaneously arise [26,27], genetic and environmental elements are almost certainly more important. The genetic factors affecting DNA methylation include the presence of structural variations, such as transposable elements (TEs) insertions/deletions (indels), chromosome rearrangements, and mutations in methylation factors [28,29], whereas important environmental conditions include temperature, drought, and other stresses [30,31,32,33], as well as in vitro culture [34,35,36].

Multiple molecular techniques have been developed to assess variation of genomic DNA methylation patterns in plants [37], with whole-genome bisulfite sequencing (WGBS; [38]) being the one displaying the highest resolution, yet the most requiring in terms of costs and computational processing. Thus, studies concerning the DNA methylation in non-model species, characterized by large and complex genomes, mostly rely on restriction-enzyme-based methods such as EpiRADseq [39].

Despite the availability of the globe artichoke genome sequence [1], as well as some resequencing data [40], and the recent identification and partial characterization of the three main classes of methyltranferases [41], global epigenomic studies have never been profiled in this species.

Here, we report an exploratory study on DNA methylation patterns on true-to-type and off-type leaves coming from three independent ‘Spinoso sardo’ plants, using the EpiRADseq method [39] with some modifications (Marconi et al., in preparation [42]). This approach allowed the identification of a set of differentially methylated loci (DML), mainly involved in flower development, photosynthesis, and regulation of reproductive processes, suggesting that further investigations on in vitro-derived epigenetics changes and their regulation might provide insights on the molecular pathways likely involved in the emergence of non-conventional phenotypes in globe artichoke.

## 2. Materials and Methods

### 2.1. Plant Material, Growth Conditions, and Samples Collection

Clones of the AGRIS3 selection of ‘Spinoso sardo’, were obtained by AGRIS agency (S’Appassiu Research Center, Cagliari, Italy) via in vitro culture of meristematic apices in growth chambers at 22 °C for 20 days under long day (16-h light, 8-h dark) photoperiods, as previously described [43]. Micropropagated plantlets were transferred in soil and grown in growth chambers under the same conditions for 20–30 days, then transplanted in open field in Cagliari (S’Appassiu Research Center), Italy (39°18 ′N, 8°55 ′E, 17 m a.s.l.). Three independent ‘Spinoso sardo’ plants, showing both true-to-type and the off-type leaves (Figure 1A) at the same age and developmental stage were chosen for sampling. Sample collection was conducted simultaneously for the three biological replicates, 3 months after open field transplantation, when the aberrant phenotype was evident and stable. Leaves from the true-to-type and the off-type branches of the same plant were collected separately, using the same tissue portions for each leaf, flash-frozen in liquid nitrogen, and stored at −80 °C. This material was used to perform both the DNA and RNA analyses reported below.

### 2.2. Library Preparation

Genomic DNA was extracted from 100 mg of flash-frozen leaves following the CTAB standard protocol [44]. Total DNA was quantified using a Qubit fluorometer 2.0 (Thermo Fisher Scientific, Waltham, MA, USA) and quality was assessed by 0.8% (*w/v*) agarose gel electrophoresis. A total of 300 ng of DNA from each sample were used for EpiRADseq library preparation. Libraries were prepared by employing the EpiRADseq protocol [39] with some modifications (Marconi et al., in preparation [42]). In brief, three true-to-type and three off-type DNA samples were used to prepare methylation-context-specific libraries. Firstly, samples underwent a double enzymatic cut (37 °C/4 h) involving the methylation-insensitive *Mse*I in combination with a methylation-sensitive enzyme, able to recognize exclusively one of the three contexts. For CG, CHH, and CHG methylation contexts, we used *Aci*I/*Mse*I, *Eco*T22I/*Mse*I, and *Fnu*4HI/*Mse*I combinations, respectively. After restriction reaction and barcoded adapter ligation (Table S1), samples were pooled and size-selected, by two purification steps based on AMPure XP beads (Beckman Coulter, Brea, CA, USA) to rescue fragments of ~300–500 bp. Pooled fragments were then biotinylated at the 5′ end through a single PCR cycle using a P1-biotynilated primer. Biotinylated DNA was mixed with Dynal M Streptavidin beads (Thermo Fisher Scientific) and bead-fragment complexes were immobilized on a magnetic base. Supernatant was removed, beads washed twice, and DNA released from beads with water. Resulting libraries followed a 10-cycles enrichment PCR reaction using adapter specific primers (Kapa Biosystems, Boston, MA, USA) and underwent a final beads purification. Library quality and fragment size were checked using Agilent Bioanalyzer (Agilent Technologies, Santa Clara, CA, USA) and the DNA was quantified by qPCR on a Step One Plus Instrument (Applied Biosystem, Foster City, CA, USA) using KAPA library quantification kit (Kapa Biosystems). Finally, libraries were sequenced with 1 × 150 bp single read chemistry on a NextSeq500 (Illumina, San Diego, CA, USA) instrument, using the High-Output Flow Cell configuration to obtain ~30 M reads.

### 2.3. Bioinformatics Analysis

Since the EpiRADseq method is based on methylation-sensitive enzymes that do not cut the genome when a cut site is methylated (and thus do not deliver a sequence read), the quantity of reads mapping to a particular locus provide evidence for the degree of methylation. Therefore, the detection of methylation status for each locus was obtained by counting methylated/un-methylated loci for each experimental sample. All the scripts used in the pipeline are reported in Supplementary Materials File S1.

#### 2.3.1. Demultiplexing and Cleaning of Reads

Raw reads were quality filtered running Sickle (https://github.com/najoshi/sickle), followed by removal of adapter sequences through Scythe (https://github.com/vsbuffalo/scythe). Reads belonging to CG and CHH libraries were demultiplexed using the *process_radtags* tool (STACKS v.1.19 package, [45]), which identified and assigned reads to each individual on the basis of 7-bp custom barcode sequences (trimmed after the analysis) and expected adjacent sequence derived from the context methylation-sensitive enzyme restriction. A manual demultiplexing of CHG reads, which were not recognizable by process_radtags, was conducted with a custom bash script mimicking the computations steps of process_radtags.

#### 2.3.2. Differentially Methylated Loci Identification

According to Reference [39], the analysis underwent a multistep approach (schematically reported in Figure S1): (i) creation of a pseudo-reference merging all loci of each library type (i.e., *Aci*I, *Eco*T22I, and *Fnu*4HI) using the clustering algorithms Rainbow and CD-HIT [46,47]; (ii) mapping the reads on the relative pseudo-reference with BWA [48], using the mem command with default parameters; (iii) counting methylated/un-methylated loci and producing count-files for each library (loci-count files). Biased loci were preliminarily filtered out, using a Student’s *t*-test *p* < 0.05, adjusted for multiple testing using the Benjamini and Hochberg method [49]. All the loci (loci-count files) showing corrected false discovery rate (FDR) values below 0.05, were used for principal component analysis (PCA) analysis and hierarchically clustering. PCA was obtained using R tools implemented in https://biit.cs.ut.ee/clustvis/. Hierarchically clustering was performed using Genesis Software (http://genome.tugraz.at/, [50]), with default parameters, maintaining all the replicate values. To provide a stringent list of statistically significant loci between true-to-type and off-type loci, in each methylation context, we used pairwise exact test of the negative binomial distribution implemented in edgeR [51] pipeline (Fisher’s exact test *p* < 0.01) applied to mean data, setting true-to-type as wild-type condition. All loci that satisfied such conditions were defined as differentially methylated loci (DML) and used for further bioinformatics analyses.

#### 2.3.3. DML Annotation

Differentially methylated loci were, then, back-aligned on the globe artichoke genome with BWA-SW command [48] and functionally annotated intersecting loci coordinates with the ‘2C’ (www.artichokegenome.unito.it, [38]) and the ‘Spinoso di Palermo’ [40] globe artichoke annotation data with BEDTools (http://bedtools.readthedocs.io, [52]). For the classification of structural features, we defined three possible genomic locations harbouring loci: (i) upstream regions (2000 bp upstream of the translational start, start codon), (ii) intragenic regions (coding DNA sequence, CDS), and (iii) downstream regions (2000 bp downstream of the translational stop, stop codon). Unknown genomic regions were further annotated using tBlastx (https://blast.ncbi.nlm.nih.gov/Blast.cgi) within Arabidopsis proteome, with default parameters and assigning putative functions to hits with e-value < 1 ‘ 10^−10^. Differentially methylated loci datasets underwent a Gene Ontology (GO) categorization using an online GO mapper suite (https://go.princeton.edu/cgi-bin/GOTermMapper), selecting a plant generic GO slim mode.

### 2.4. RNA Isolation and Gene Transcription Analysis

RNA was extracted from 100 mg of flash-frozen leaves with the RNeasy Plant Mini Kit (Qiagen, Hilden, Germany) following the manufacturer’s instructions. RNA samples were retro-transcribed in cDNA using the High-Capacity cDNA Reverse Transcription Kit (Applied Biosystem). cDNA of true-to-type and off-type samples were tested on Step One Plus Real Time PCR (Applied Biosystem) and the transcript level was calculated using the ΔΔCT method. As housekeeping genes, actin (XM_025103545) and elongation factor 1 (EF1, XM_025112661) were chosen for their stability of transcript level [53]. RT-qPCRs were performed in three biological and three technical replicates using 2X Power SYBR**^®^** Green PCR Master Mix (Applied Biosystem). PCR conditions comprised an initial incubation of 95 °C/10 min, followed by 35 cycles of 95 °C/15 s and 60 °C/45 s. Melting curve analysis was performed at the end of amplification. Standard curves were analyzed using Step One Plus software. Primers used in this analysis were designed on the ‘Spinoso di Palermo’ re-sequenced genome [40] using Primer 3 v.4.1.0 software (http://bioinfo.ut.ee/primer3/) and their sequences are reported in Table S2. A *t*-test was performed to assess the statistical significance of different transcript levels between the three true-to-type and three off-type replicates.

### 2.5. miRNA Target Genes Analysis

Genic DML were subjected to a miRNA target analysis using the psRNATarget (http://plantgrn.noble.org/psRNATarget) tools using the *C. cardunculus* specific miRNA database (miRBase release 21) and default conditions (i.e., schema V2, 2017), considering a maximum expectation of 5 and inhibition of translation for mismatches in the 10th to 11th mature miRNA nucleotides. A GO enrichment analysis of miRNA target genes was implemented using the enrichment term engine (GO terms, KEGG pathways/INTERPRO domain) implemented in STRING (https://string-db.org), with false discovery rate <0.01.

### 2.6. Accession Code

Sequencing raw data were deposited in Sequences Read Archive under the accession number SRP150592.

## 3. Results

### 3.1. Sequencing Results

Illumina sequencing generated 27.28 M raw reads, which were de-multiplexed in the three methylation-context-specific original libraries. In particular, three datasets containing 14.92 M, 8.58 M, and 2.27 M reads were populated for the CG (AciI library), CHH (EcoT22I library) and CHG (Fnu4HI library) context, respectively. Approximately 3.5% of reads were identified as low quality after the process of trimming and cleaning and were removed (Table S1). The remaining 26.3 M reads (96.5%) were kept for mapping and subsequent bioinformatics analyses (Table S1).

### 3.2. Differentially Methylated Loci

**Figure 2:**
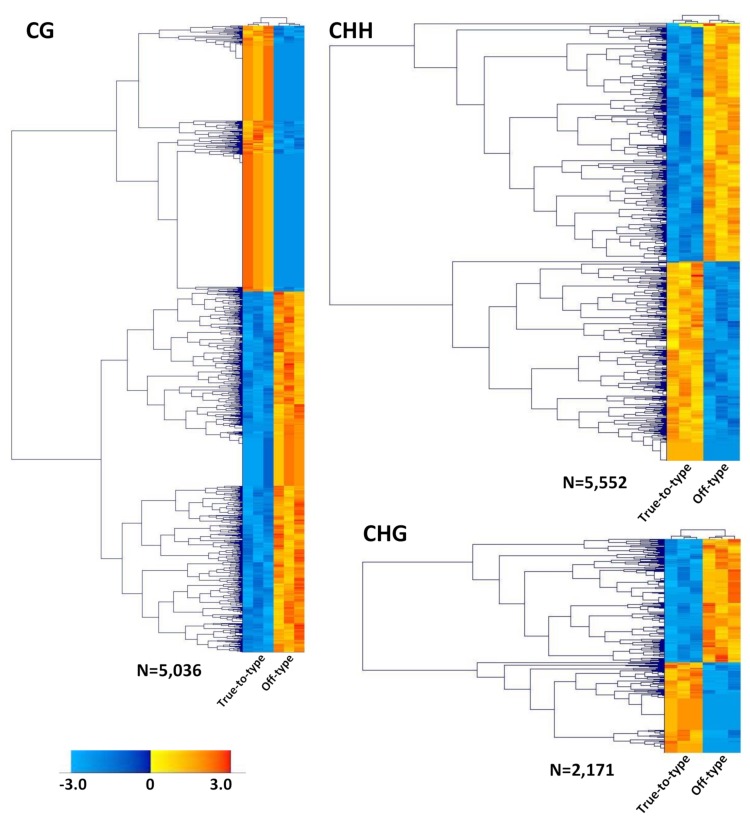
Heatmaps of CG, CHH, and CHG differentially methylated loci. Normalized loci, expressed as log2 (Fold Change) of the relative count numbers, from three replicates of true-to-type and off-type samples, were clustered using hierarchical average linkage clustering and Euclidean distances (*p* ≤ 0.05). Columns and rows represent samples assayed (true-to-type on the left, off-type on the right) and differentially methylated loci (DML), respectively. For each methylation context two main clusters are distinguishable, showing an opposite methylation trend between true-to-type and off-type phenotypes. Color-code of differential methylation state is reported on the bottom left, resulting in values between −3 (=maximum level of methylation; blue) and 3 (=no methylation; orange).

**Figure 3:**
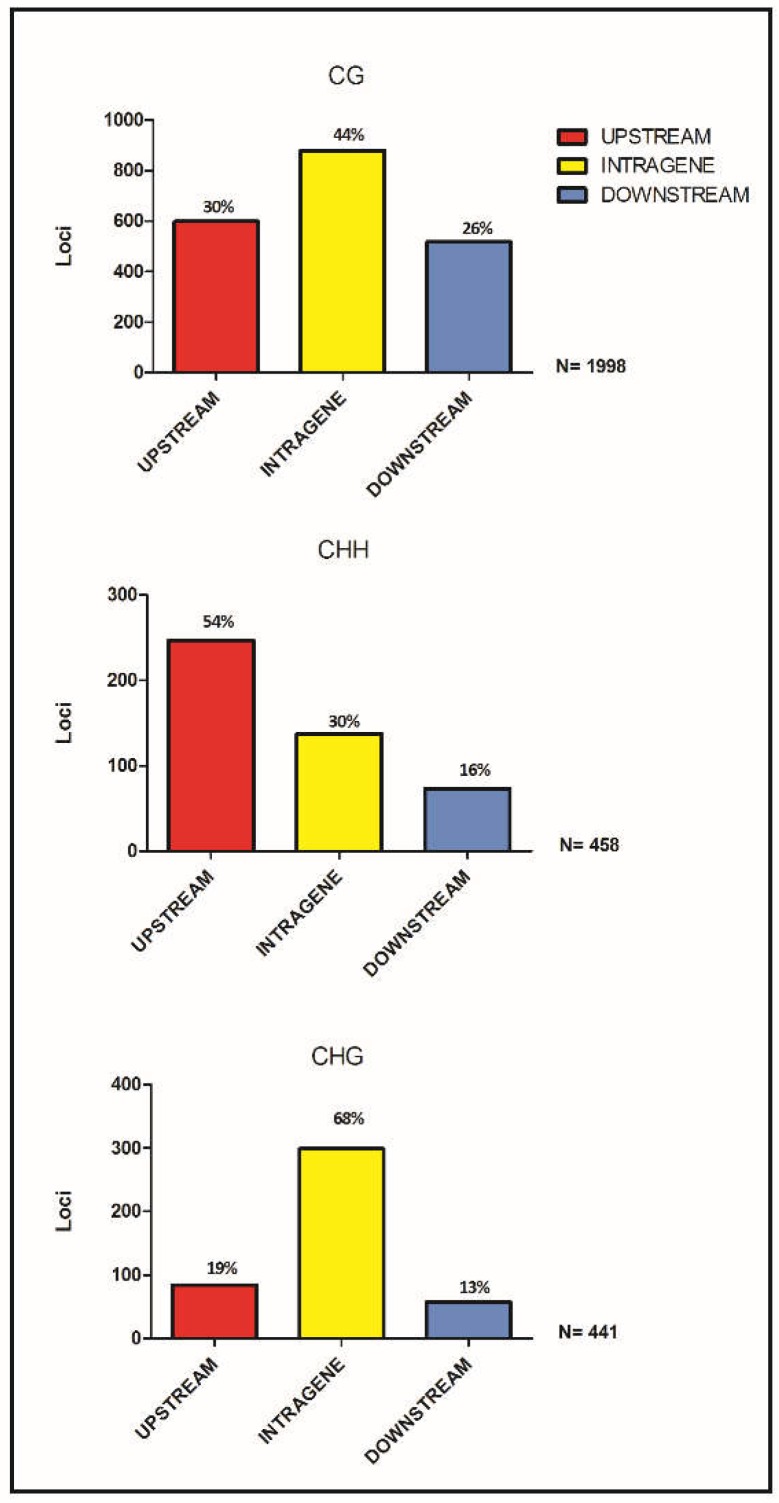
Genome-wide distribution of DML. Different localization of differentially methylated loci in the three contexts of methylation is highlighted in the bar plots. N is the library-specific number of DML obtained in the bioinformatics analysis. Upstream regions are identified in a window of 2000 bp upstream the translational start (start codon), intragenic regions overlap with coding DNA sequence (CDS), downstream regions refer to a window of 2000 bp downstream the translational stop (stop codon).

For each context-specific set of loci, a pseudo-reference was constructed [39]. Reads from specific libraries were back-aligned on each pseudo-reference with an average mapping efficiency of 97% for CG, 92.1% for CHH, and 98.4% for CHG. Overall, 121,694 loci in CG, 172,934 in CHH, and 117,195 in CHG contexts were assessed and the number of high-quality reads mapped to each locus was counted. Biased loci were filtered-out and 5036 CG, 5552 CHH, and 2171 CHG high-quality loci were retained for further analyses. To detect consistent shift in methylation patterns between true-to-type and off-type samples, PCA and hierarchical clustering analyses of filtered DML were performed, considering all the replicates. PCA for all the three contexts graphically showed the separation between samples based on methylation levels of DML (Figure S2). The first component (PC1) accounted for 82.5%, 79.6%, and 83% of the total variance, for CG, CHH, and CHG, respectively, and clearly discriminated off-type from true-to-type replicates indicating that methylation changes occurs between these two contrasting phenotypes. The three resulting heatmaps displayed a consistent methylation trend between true-to-type and off-type biological replicates (Figure 2). In CG and CHH contexts, an almost even distribution between hypo (~47%, 2367 out of 5036, and 2601 out of 5552, respectively) and hyper (~53%, 2669 out of 5036, and 2951 out of 5552) methylated loci was observed in true-to-type samples compared to the off-type ones. Differently, in CHG, roughly 40% of DML showed a hypo-methylated (862 out of 2171) and ~60% a hyper-methylated (1309 out of 2171) behavior in true-to-type samples compared to the off-type ones (Figure 2). The edgeR pipeline provided a stringent list of statistically significant loci for each context of methylation; in particular, 1998 DML in CG, 458 DML in CHH, and 441 DML in CHG were observed. Using DML sequences and coordinates extrapolated from the main clusters of each heatmap, we observed that loci detected in CG and CHG were mainly present in intragenic regions (44% and 68%, respectively), while the majority of CHH loci were in upstream regions (54%), where gene promoters are normally located (Figure 3).

### 3.3. DML Annotation and GO Categorization Analysis

Functional annotation of DML was conducted on the bases of the available ‘2C’ globe artichoke reference genome (www.artichokegenome.unito.it). The DML located within coding regions were 960 of which 720 out of 1998 in CG, 88 out of 458 DML in CHH, and 152 out of 441 DML in CHG methylation contexts (Table S3).

**Figure 4:**
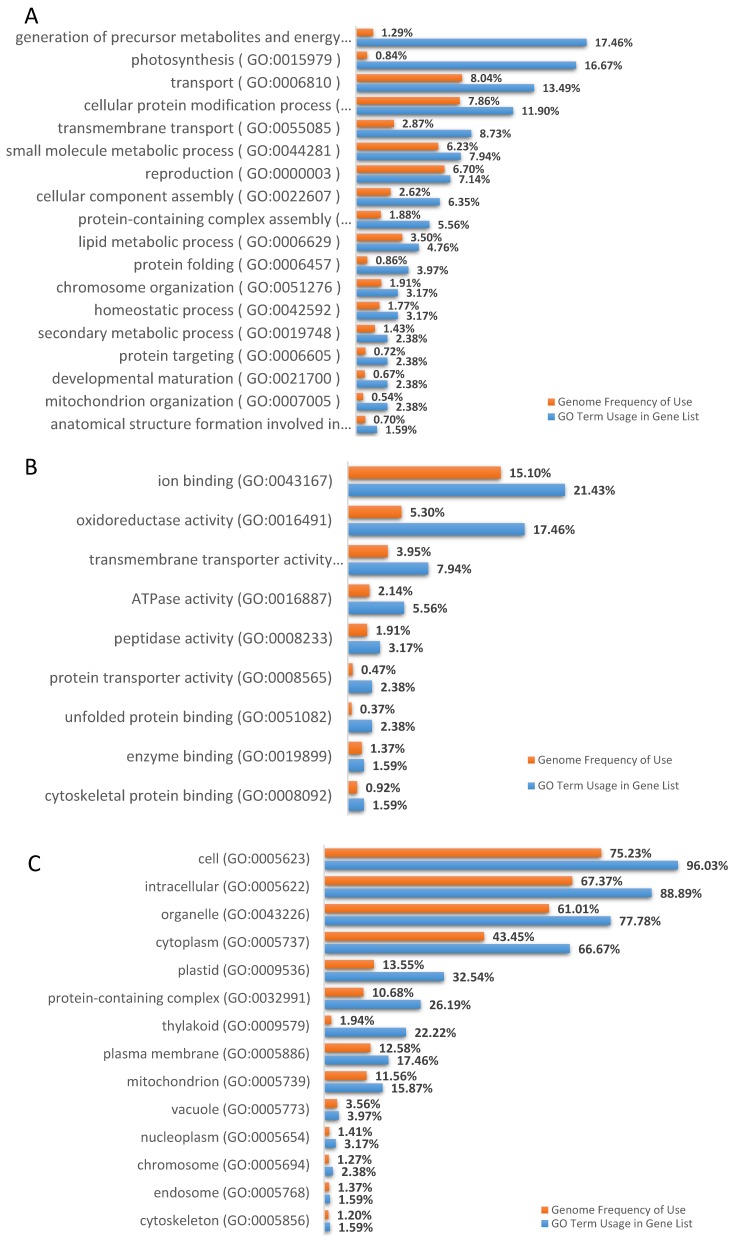
Gene ontology (GO) categorization of genic DML—(**A**) biological processes; (**B**) molecular functions; (**C**) cellular components. The blue bars indicate input, represented by all true-to-type and off-type loci, while the orange bars indicate background genome genes.

Gene ontology (GO) analysis of all coding DML revealed different GO terms (Figure 4). Among biological processes (BP), remarkable GO terms were observed in: photosynthesis, transmembrane transport, reproduction, protein folding, chromosome organization, protein targeting, developmental maturation, and anatomical structure formation involved in morphogenesis. With respect to molecular functions (MF), prevalent GO terms were: ion binding, oxidoreductase activity, transmembrane transporter activity, ATPase activity, peptidase activity, unfolded protein binding, protein transporter activity, and cytoskeletal protein binding. For cellular components (CC), the most relevant GO terms were: cytoplasm, plastid, protein-containing complex, thylakoid, plasma membrane, mitochondrion, vacuole, nucleoplasm, chromosome, cytoskeleton, and endosome.

### 3.4. Gene Transcriptional Analysis

A subset of interesting genes for each methylation context was selected for additional transcriptional analyses. We focused on 14 genes, which showed a perfect restriction site in the locus sequence and are involved in epigenetic and transcriptional regulation of organ and developmental maturation and plant reproduction (e.g., GO:0021700, GO:0048646, and GO:0000003), processes likely fitting to our contrasting phenotypes [54,55,56,57,58,59,60,61,62,63,64,65,66,67,68] (Table 1). In order to study whether the transcription of differentially methylated genes varies between true-to-type and off-type biological replicates, qPCRs on the cDNA derived from the same leaves used for the EpiRADseq analysis were performed. Five genes, namely ATX1, SCAR2, TPL, TSO1 and MED33A, showed a statistically significant difference in transcription between true-to-type and off-type replicates (Table 2 and Figure S3).

### 3.5. microRNAs Target in Silico Analysis

**Figure 5:**
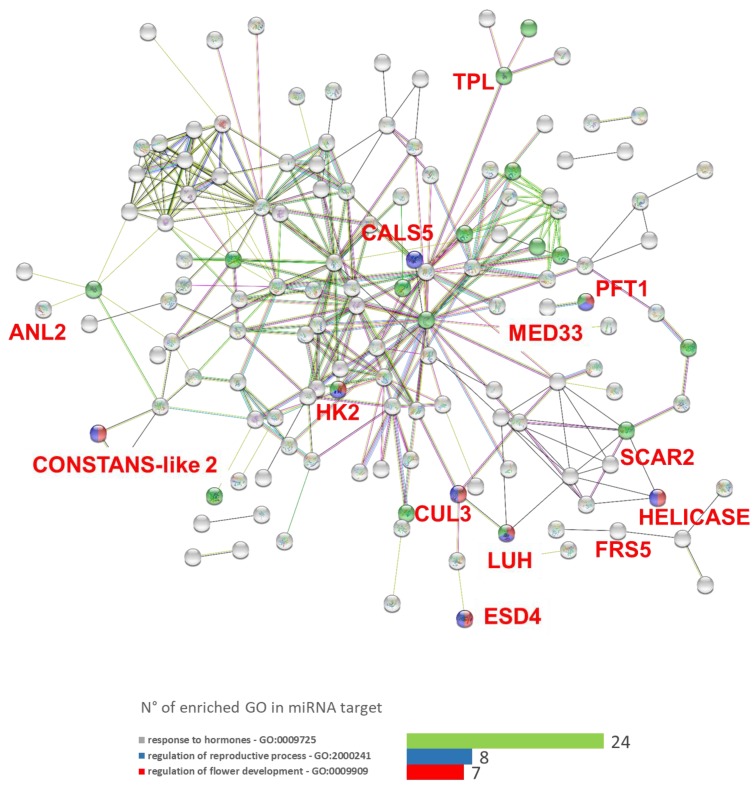
Interactome graph of the DML miRNA target and selected GO enrichments. Enrichments are highlighted as red/blue/green dots, as shown in the legend.

The analysis revealed that some coding differentially methylated loci were either putative miRNA targets or secondarily involved in miRNA pathways (S4 Table). In details, 442 putative miRNA-miRNA target associations were predicted, consisting of 236 unique target genes and 59 unique miRNAs. To investigate any relations among those genes found to be jointly differentially methylated and putative miRNA target, an interactomic map was established (Figure 5). Regarding the interactions observed in the network made of DML putative target of miRNA, a number of 205 nodes and 313 edges were observed (average node degree = 3.05). Considering the value of 211 as the expected number of edges for 205 nodes, this network had significantly more interactions than expected (PPI enrichment *p*-value = 4.13 × 10^−11^). A sub-network was observed, related to (1) primary metabolic process (GO:0044238, 77 genes, Figure 5), mostly involved in photosynthesis. In addition, we observed some enrichments, likely coherent with phenotype: (1) regulation of flower development (GO:0009909, 8 genes); (2) regulation of reproductive process (GO:2000241, 7 genes). To prove that the enrichment above reported was not an effect of bioinformatics artefacts and rearrangements, a random sampled set of sequences was assayed as well, without producing any enrichment. Among all the putative miRNA targets revealed by psRNA target analysis, the statistically significant genes for transcriptional analysis (ATX1, SCAR2, TPL, TSO1 and MED33A, Table 2) showed to be in silico targets of miRNAs involved in plant developmental processes such as flowering transitions, floral meristem formation, or cell differentiation (e.g., miR167, miR160, miR172, and miR396, Table 2, Table S4).

## 4. Discussion

Vegetative multiplication is the privileged method for the propagation and the maintenance of élite genotypes to preserve their characteristic traits. However, it has been well established that in vitro techniques favor the emergence of unwanted variability in many species [69,70]. Some examples of this phenotypic variability are the ‘mantled’ flowers in oil palm [71], the bushy plants in gerbera [72], the dwarf ‘choke-throat’ or giant plants in banana [73,74], and the variation of the leaf shape in begonia [75].

Globe artichoke ‘Spinoso sardo’, is an elite varietal type, which has been recently registered as a PDO product (namely ‘Carciofo Spinoso di Sardegna’, IT/PDO/0005/0687), representing the most profitable income for Sardinian rural economy. The in vitro culture of meristematic apices is a common method used to obtain systemic pathogen-free globe artichoke plants and a higher clonal multiplication rate compared to conventional agamic multiplication. Although, in species such as sweet potato [76] and cassava [77] meristem micropropagation has often been reported as the technique that allows growers to obtain plants more faithful to the parental plant phenotype, in globe artichoke, a phenotypic variability already described [78,79] occasionally happens. Indeed, in ‘Spinoso sardo’ production, some plants show two different phenotypes: the off-type, characterized by highly pinnate-parted leaves and a late budding of the inflorescence, in contrast with the desirable true-to-type phenotype. This phenomenon is unpredictable and may occur in micro-propagated plants in in vitro phase as well as later, in open fields. Phenotypic, cytological, and biochemical deviations expressed in the progeny of plants regenerated from in vitro culture are known as ‘somaclonal variation’ [80].

Genetic (i.e*.,* single base-pair changes, chromosome deletions, translocations, insertions, and changes in ploidy) as well as epigenetic (e.g*.,* DNA methylation) alterations are often associated with in vitro propagation. Both these alterations are genotype-dependent [81] and influenced by the explant source, as well as by the stressful in vitro environmental conditions (i.e., high relative humidity, culture period length, high concentrations of sugars and plant growth regulators, and low light availability, [18]). Even though there is no doubt that DNA sequence mutations are the primary cause of growth defects, local DNA methylation variants and correct transmission of epigenetic marks are crucial for plant development [36]. Alterations in global methylation patterns in in vitro culture have been reported [82], and reflect the adaptation process of cells to a different environment which includes the response to signals that may trigger switches in the developmental program. So far, in ‘Spinoso sardo’ factors like the number of subculture cycles, the sampling period and the relative size of offshoots (containing meristem apex) have been investigated to study their potential correlation with the off-type phenotype emergence [43,83]. Results showed that the sampling period did not influence the number of aberrant phenotypes [83]. On the other hand, the percentage of off-type plants grew with the increase in the number of subcultures and the reduction in the size of the explant [80]. The unpredictability of the phenomenon seems in line with the hypotheses of either genetic or epigenetic changes as a cause of the off-type plants. However, morphological variance in each plant does suggest an epigenetic cause (rather than genetic). Baránek and collaborators [84] found consistent differences in methylation sensitive amplification polymorphism (MSAP) profiles of daughter plants recovered from field cuttings and micro-propagated nodal segments of two grapevine varieties. Kitimu and collaborators [70] combined MSAP and methylation sensitive GBS (msGBS; [85]) to survey for DNA methylation variations between samples of cassava (*Manihot esculenta*) taken from field-grown cuttings and those recovered from meristem culture. Therefore, this study was aimed at understanding whether DML could be the basis of the phenotypic difference between true-to-type and off-type. Due to the complexity of the globe artichoke genome and the lack of epigenetic data for this species, here we employed EpiRADseq to gain a general picture of globe artichoke genome-wide DNA methylation. Despite their limited resolution, methylation assessments based on restriction-enzyme have been largely performed in the last decade and still represent the most used and feasible strategies to study DNA methylation patterns in wild non-model plants [86,87,88]. In this study, EpiRADseq protocol [39] was employed with some modifications (Marconi et al., in preparation [42]). In particular, to study the CG context, the commonly used *Hpa*II enzyme [39,86] was replaced with *Aci*I, which is completely inhibited in the presence of methylated cytosine in its restriction site. Furthermore, the use of *Fnu*4HI enzyme, which recognizes the 5′-GCNGC-3 ′site, and *Eco*T22I, which has a 5′-ATGCAT-3′ site, allowed us to study the methylation in the CHG and CHH contexts, respectively. Our approach allowed the investigation of DML between true-to-type and off-type sets of samples in the plant-specific three-methylation contexts, where they showed differences in both genomic position (Figure 3) and correspondence with DNA methylation levels. Interestingly, most of them belong to genes directly or indirectly involved in reproductive processes and organ development and morphogenesis (Figure 4), which are largely affected by environmental stresses and DNA methylation switches.

Methylation in all contexts is located within transposable elements, which are nearly ubiquitously methylated in land plant genomes to guarantee biological integrity and transcriptional homeostasis [89]. In addition to transposons, DNA methylation frequently occurs in active plant genes [90]. Although there is evidence that methylation is shaped by transcriptional activity in *Arabidopsis* [91], how this synergistic interplay of DNA methylation and transcription takes place in non-model species has not been explored yet. Bioinformatics analysis highlighted a set of 14 candidate genes (Table 1) harboring significant differences in methylation level between true-to-type and off-type samples, which were thus used to assess transcriptional analysis. Five genes (TPL, SCAR2, MED33A, ATX1 and TSO1, Table 2) showed a statistically significant difference in transcriptional level between the true-to-type and the off-type biological replicates, with a decrease in the transcriptional activity of off-type replicates (Figure S3). However, cytosine methylation status is different among those genes. Namely, SCAR2, TSO1, and MED33A were hypo-methylated in the restriction site, recognized by the restriction enzymes used in this analysis, in true-to-type samples compared to off-types, while ATX1 and TPL were hyper-methylated (Table 1). All the differentially methylated loci for these were located in exons (Table 1), thus it was difficult to predict their transcriptional activity, since the effects of DNA methylation in gene bodies (GbM methylation) still remains cryptic [90]. The unknown consequences of GbM and the possible intervention of different layers of regulation other than DNA methylation might be some plausible explanations to the observed absence of correlation between methylation status and transcriptional activity of the candidate genes.

The absence of statistical significance in the other genes analyzed might be due to tissue-culture stress that induces a high spectrum of phenotypic and molecular changes in plant regenerants [34,75]. Moreover, it is difficult to minimize the biological variability in aberrant plants due to non-controlled conditions in open field production.

Although growth chambers and greenhouses offer the advantage that cultivation conditions can be set to the experimental needs and repeatedly applied to check reproducibility of observations, results obtained in a controlled environment often display low correlation with field assays [92,93]. Moreover, even in climate-controlled phyto-chambers, which are supposed to provide the best possibility to dissect plant traits with good reproducibility, micro-environmental variations can highly affect experimental reproducibility, as reported for the model plant *Arabidopsis thaliana* [94]. Because the purpose of our research was to set the bases to untangle the possible triggers of off-types appearance after transplantation, a field experiment was necessary. In the attempt to reduce variability within biological replicates, several efforts to maintain uniformity of the sampled material and an accurate selection of plants sharing the same age and developmental stage were applied. Our data showed that, although sensitive to environmental variations, DNA methylation changes are coherent in the three biological replicates of true-to-type and off-type samples (Figure 2), demonstrating the reliability of our experimental setup. On the other hand, transcriptional analysis displayed a high degree of variability as only 5 genes (out of the 14 tested), showed a reproducible expression level within replicates, suggesting that other regulation mechanisms might interfere with the transcriptional activation of the candidate genes selected (e.g., histone modification, miRNAs, etc.)

We also evaluated another level of epigenetic control likely affecting the ‘Spinoso sardo’ aberrant phenotype, focusing on microRNAs (miRNAs) regulation. miRNAs are involved in both transcriptional and epigenetic regulations, which are organized by a multiple combination of interfering pathways [95,96,97,98]. A fine-tuned balance between microRNAs and their target genes is fundamental to promote leaf differentiation and plant development [99,100]. Due to their impact on gene expression mechanisms, numerous studies arose with the purpose of elucidating miRNAs specificity and their function in plant growth [101,102]. Our results showed some in silico connections between DML and miRNA pathways involved in the machinery of flowering time and plant development (Table 2, Table S4). This was also supported by the interactomic analysis highlighting many links between candidate genes in the network, also supported by the protein–protein interaction (PPI) enrichment (average node degree = 3.05, *p*-value = 4.13 × 10^−11^), which appeared higher than expected.

As an example, we highlighted a DML present in TPL (TOPLESS) [66] which, in globe artichoke, is a putative target of the leaf shape-regulatory miR396 [103]. ATX1 is a histone H3K4 methyltransferase responsible for floral organ development [56,57]; we observed that, in our data, it is a putative target of miR172 and miR167 whose role in flowering has been fully demonstrated [104]. There are also evidences about the direct influence of miR156/157, which regulation is often combined with miR172, on ATX1 [105], alluding to their potential contribution to flowering time defects in globe artichoke. Moreover, SCAR2 is, in silico, targeted by miR319 which is normally associated to the TCP transcription factors family involved in organ and flower development [106], suggesting that a similar regulation might take place in SCAR2 molecular pathway. The aforementioned genes also reflect the most significant difference in transcription level between true-to-type and off-type plants (Figure S3).

Once more, due to the reduction-of-complexity nature of EpiRADseq, we cannot propose the causality of changes in methylation of some miRNA-related genes and the phenotype observed in the field, but merely show a likely correlation between methylation target sites and ‘Spinoso sardo’ developmental transitions. Although an accurate experimental validation is needed in order to elucidate the biological significance of our findings, these in silico data open a new possible scenario about a dual DNA methylation and miRNA-mediated regulation of plant development in ‘Spinoso sardo’ ecotype.

## 5. Conclusions

We demonstrated that the application of the EpiRADseq protocol on ‘*Spinoso sardo’* genotypes allowed the identification of different methylation patterns between true-to-type and off-type leaves sampled on the same plant. The functional annotation of DML allowed us to identify candidate genes coding for proteins involved in flower development and its regulation, maintenance of epigenetic modifications, and vegetative development. Further research focused on biological mechanisms and technological advances in the coming years are likely to broaden our understanding on which molecular and environmental cues elicit the emergence of off-type plants. These will increase the opportunities to monitor and control crop epigenomes [107] for the stable improvement of agricultural traits of élite cultivars.

## Figures and Tables

**Table 1:** Features belonging to a subset of differentially methylated loci. Loci are characterized by context, hypo-methylation (‘−‘) or hyper-methylation (‘+’), genomic position, gene ID, and putative function.

*Locus* epi-RADseq	Context	*True-to-type* *locus*	*Off-type* *locus*	Gene name	Genomic position	Gene ID	Function	Ref.
E8364_L143	ACI	-	+	IDM1	exon	Ccrd_v2_02819_g02	epigenetics regulation	[51]
E245746_L143	FNU	-	+	AHK2	exon	Ccrd_v2_04778_g02	plant organ size, flowering time and plant longevity	[52]
E232952_L143	ACI	+	-	ATX1	exon	Ccrd_v2_10262_g06	regulation of flowering	[53,54]
E258509_L71	ECO	-	+	HST	exon	Ccrd_v2_10596_g06	regulation of morphogenesis	[55]
E421706_L143	ACI	-	+	COL2	exon	Ccrd_v2_11089_g07	circadian-clock dependent regulation of flowering time	[56]
E225812_L143	FNU	-	+	ARF19	exon	Ccrd_v2_11769_g08	lateral organ development	[57]
E243304_L143	FNU	-	+	TSO1	exon	Ccrd_v2_12311_g08	flower organogenesis and development	[58]
E339914_L127	ECO	-	+	SCAR2	exon	Ccrd_v2_13193_g09	regulation of plant morphogenesis and flowering	[59]
E243912_L143	FNU	-	+	MED33A	exon	Ccrd_v2_13680_g09	transcriptional regulation of flowering time	[60]
E255497_L143	ACI	-	+	JMJ25	exon	Ccrd_v2_19643_g13	epigenetic regulation of development	[61]
E172485_L143	FNU	-	+	HAP2	exon	Ccrd_v2_22114_g15	plant reproduction organs	[62]
E256023_L143	ECO	+	-	TPL	exon	Ccrd_v2_22902_g16	regulation of flowering	[63]
E244185_L143	ACI	+	-	ANL2	exon	Ccrd_v2_26026_scaffold_1939	epigenetic regulation of flowering time	[64]
E245348_L143	FNU	-	+	FRS5	exon	Ccrd_v2_10161_g06	light-induced regulation of plant development	[65]

**Table 2:** Transcriptional and miRNA target analyses of a subset of differentially methylated genes. For each gene transcription level, miRNAs and presence in the interactomic analysis are reported.

Gene Name	Transcription Level	miRNA Target	Interactome miRNA Target
ATX1	Down-expressed in off-type	Cca-miR6107, cca-miR167, cca-miR172	-
SCAR2	Down-expressed in off-type	cca-miR319	yes
TPL	Down-expressed in off-type	Cca-miR396b	yes
TSO1	Down-expressed in off-type	Cca-miR395b, cca-miR395c, cca-miR6116-5p	-
MED33A	Down-expressed in off-type	cca-miR160a	yes

## Supplementary Materials

The following are available online at https://www.mdpi.com/2073-4425/10/4/263/s1, Figure S1: Flow-chart of bioinformatics pipeline. The flowchart underlies the key points of sequence processing. Arrows indicate the sequential steps that raw reads underwent during the analysis. Programs (in white), input (in light-blue), and output (in orange) files, are also indicated in the legend (top right), Figure S2: PCA of relative methylation level at DML for each sequence context, CG, CHH, and CHG. Numbers in brackets indicate the fraction of overall variance explained by the respective component (PC1 and PC2), Figure S3: Transcription analysis. qPCR evaluation of transcriptional activity was carried out for five selected genes belonging to the three methylation contexts: ATX1 (CG), SCAR2 and TPL (CHH), MED33A and TSO1 (CHG). White bars represent true-to-type replicates, while off-type are indicated in grey. Error bars represent SD (n = 3). All genes reported show statistically significant differences in transcription levels between the three true-to-type and off-type replicates based on *t*-test (*p* ≤ 0.05), Table S1: Mapping statistics. For each sequenced sample indicated in Column 1, numbers of raw reads, cleaned-trimmed reads, uniquely and multi-mapped reads and duplicates removed are reported (Columns 2–5, respectively), Table S2: List of primers used for qPCR analysis, Table S3: Differentially methylated loci, present in coding sequences, output of edgeR analysis. Both genes ID, referred to globe artichoke reference genome, and EpiRADseq ID notation, referred to the DML, are reported for the three methylation contexts, Table S4: List of putative miRNA targets, File S1: Bioinformatics pipeline for the sequence reads analysis.
