# Supplementary figures and images for: Analysis of DNA Methylation Patterns Associated with In Vitro Propagated Globe Artichoke Plants Using an EpiRADseq-Based Approach
Source: Genes (Basel). 2019 Apr 1;10(4):263. doi: 10.3390/genes10040263 (PMC6523903; doi:10.3390/genes10040263)

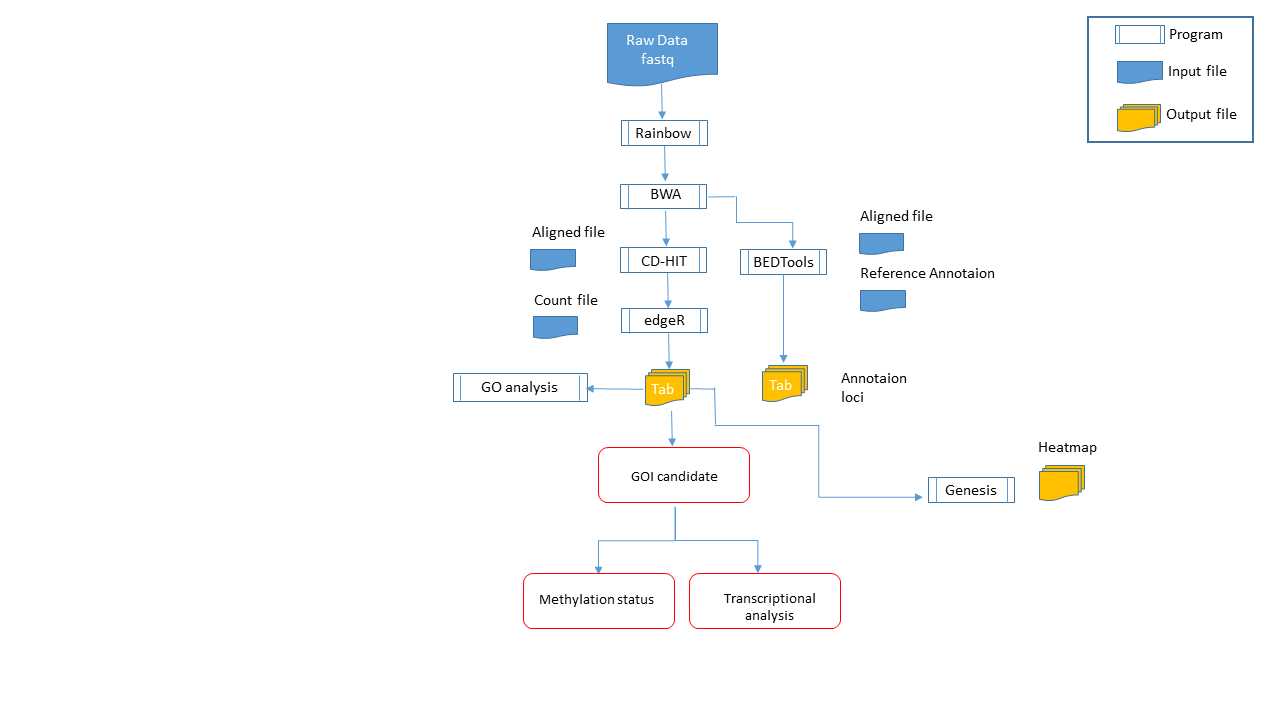

Supplement: Supplementary file 1 [file genes-10-00263-s001.zip › Figure S1.tif]

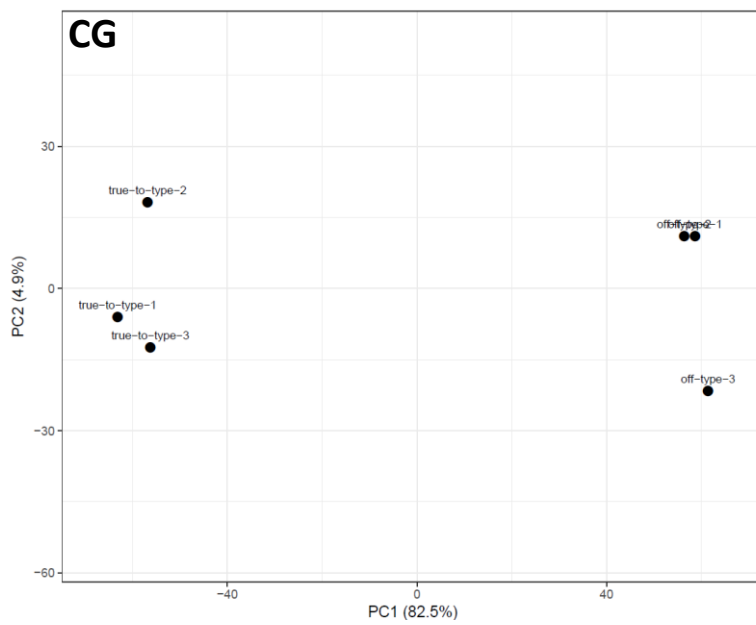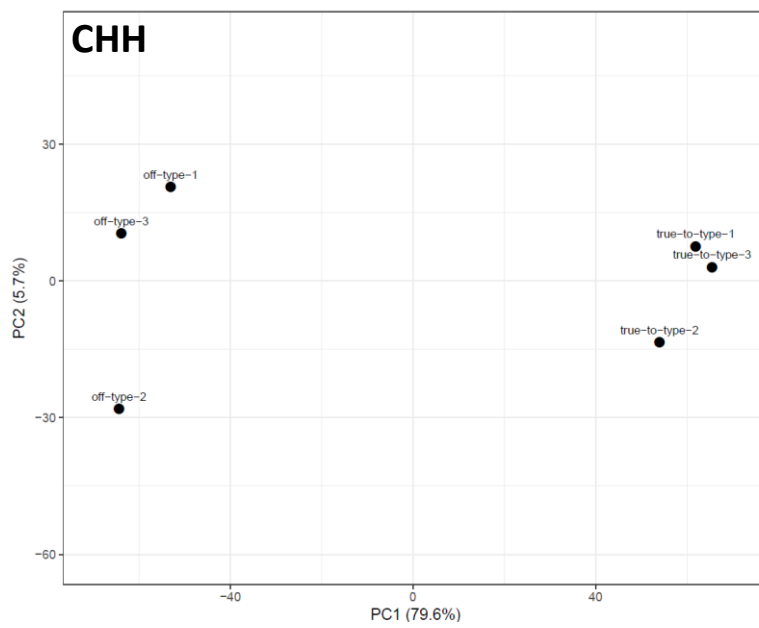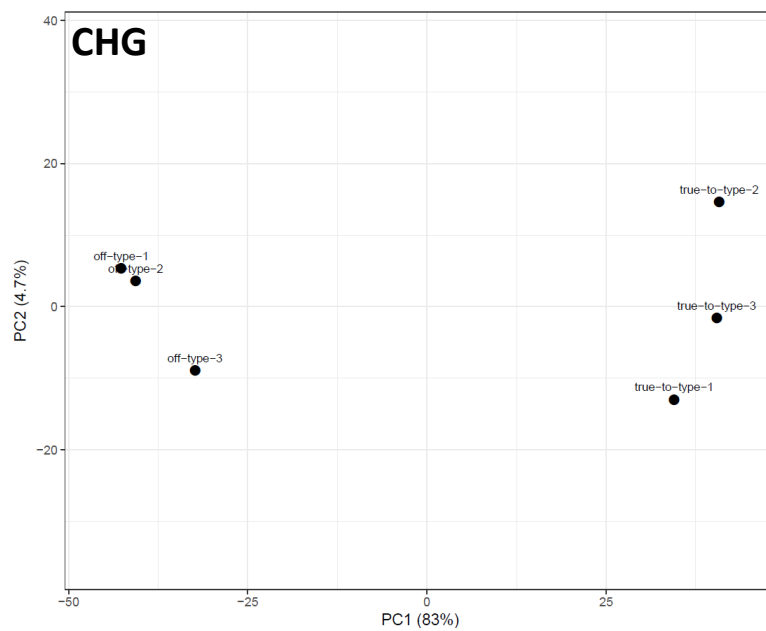

Supplement: Supplementary file 1 [file genes-10-00263-s001.zip › Figure S2.pdf]

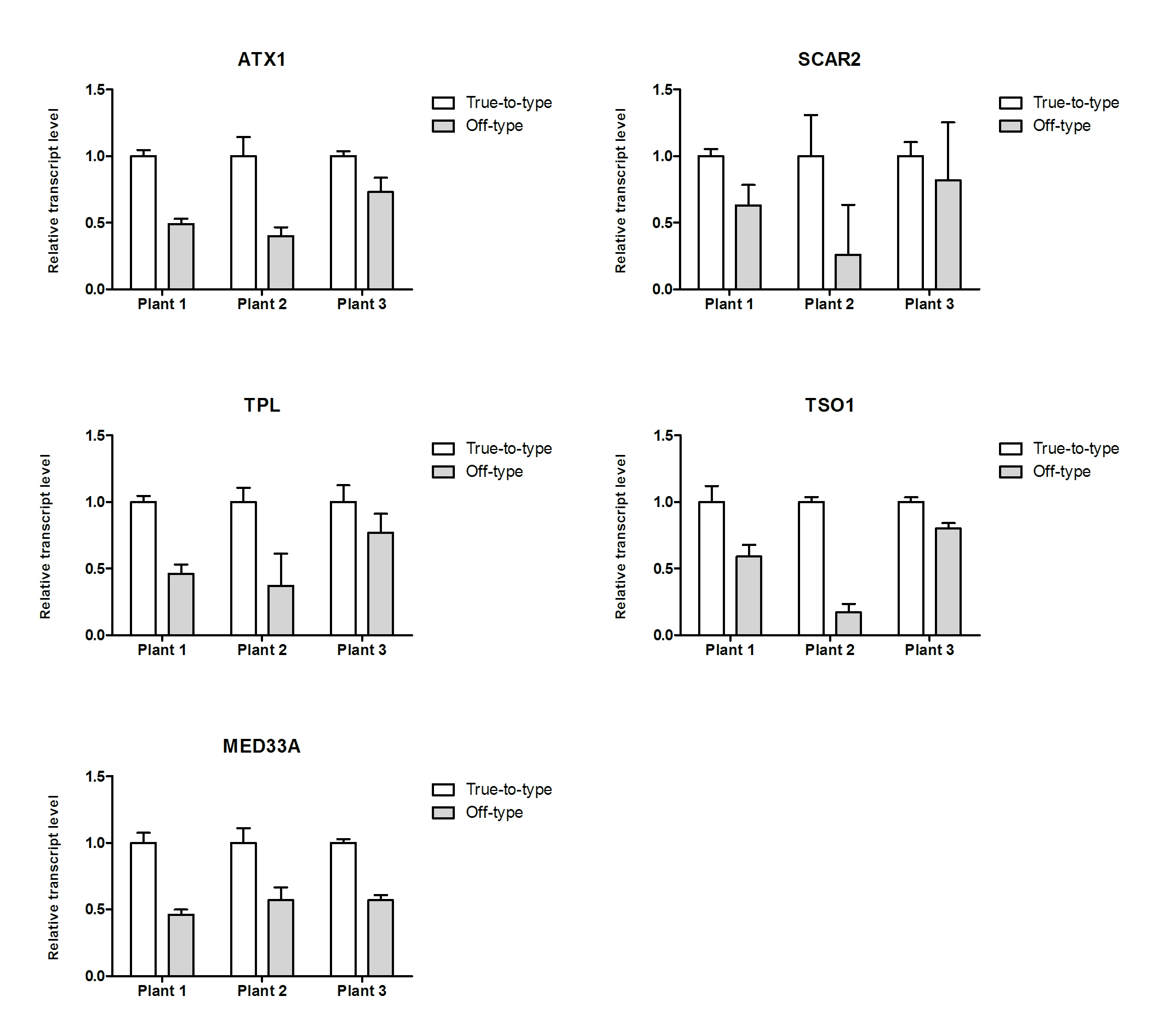

Supplement: Supplementary file 1 [file genes-10-00263-s001.zip › Figure S3.tif]
